# Skipping the co-expression problem: the new 2A "CHYSEL" technology

**DOI:** 10.1186/1479-0556-2-13

**Published:** 2004-09-13

**Authors:** Pablo de Felipe

**Affiliations:** 1 Centre for Biomolecular Sciences, School of Biology, Biomolecular Sciences Building, University of St. Andrews, North Haugh, St. Andrews KY16 9ST, Scotland, UK

## Abstract

The rapid progress in the field of genomics is increasing our knowledge of multi-gene diseases. However, any realistic hope of gene therapy treatment for those diseases needs first to address the problem of co-ordinately co-expressing several transgenes. Currently, the use of internal ribosomal entry sites (IRESs) is the strategy chosen by many researchers to ensure co-expression. The large sizes of the IRESs (~0.5 kb), and the difficulties of ensuring a well-balanced co-expression, have prompted several researchers to imitate a co-expression strategy used by many viruses: to express several proteins as a polyprotein. A small peptide of 18 amino acids (2A) from the foot-and-mouth disease virus (FMDV) is being used to avoid the need of proteinases to process the polyprotein. FMDV 2A is introduced as a linker between two proteins to allow autonomous intra-ribosomal self-processing of polyproteins. Recent reports have shown that this sequence is compatible with different sub-cellular targeting signals and can be used to co-express up to four proteins from a single retroviral vector. This short peptide provides a tool to allow the co-expression of multiple proteins from a single vector, a useful technology for those working with heteromultimeric proteins, biochemical pathways or combined/synergistic phenomena.

## Introduction

For the last 20 years, the gene therapy field has centred many of its efforts on finding ways to deliver a therapeutic gene to certain target cells in order to produce a therapeutic result. It was soon clear that it was necessary to deliver at least two genes, because a reporter/marker gene was needed in order to track the expression of the therapeutic gene (normally not easy to detect). There has been a large increase in vector development during these years, with the appearance of many new viral and non-viral vectors. However, since the late 1980s, few improvements have been made 'inside' those vectors. The linkage of the two genes of interest (therapeutic and reporter) has remained the same. The different strategies known for co-expression were reported during the 1980s -splicing, multiple promoters, fusions, reinitiation and IRESs-, at the same time that the first gene therapy experiments were being performed (for a review [1]). During the 1990s, nearly all those strategies were abandoned in favour of the IRESs. In bicistronic mRNAs bearing an IRES sequence, the first cistron is translated by scanning ribosomes that enter via the 5' end. The cloning of an IRES sequence downstream of the first cistron, allows the internal entry of ribosomes that translate the second cistron. As each cistron is translated from a different translational initiation event, both translations are uncoupled, and the proteins are not obtained in an equimolecular proportion ("imbalance") leading to a large excess of the first protein.

The drive to co-express more than two genes, opening the door to therapies for muti-gene deficiencies, was halted by the inability of vector technology to guarantee a reliable co-expression. Nevertheless, IRESs were the first strategy that met with some success, and several polycistronic vectors able to co-express up to 4 genes were developed during the 1990's [2]. However, two main problems blocked the successful use of large and complex polycistronic vectors: the large size and imbalance of most IRESs which makes it very difficult to predict the level of expression of the downstream cistron [3].

This commentary discusses several recent publications that use self-processing polyproteins as a novel strategy for co-ordinated co-expression of several genes.

## Discussion

Although gene therapy has employed the viruses as vectors, the co-expression strategies previously described have not taken advantage of the dominant ways in which viruses achieve co-expression in cells. It is the polyprotein strategy that many viruses use to co-express most of their proteins, or even all of them (as in picornaviruses). Not surprisingly, this strategy is indeed used by cells, although not very often, in particular for the co-ordinated secretion of different proteins and peptides. Recently, several groups have been trying to utilize this co-expression strategy. One of the possibilities is to introduce the target site for a cellular proteinase between two cistrons cloned in frame forming a single open reading frame (ORF; [4]). In this way the polyprotein is synthesized as a fusion protein that post-translationally is proteolytically cleaved to yield the discrete proteins of interest. Unfortunately, this strategy has several practical difficulties: (1) the polyprotein to be cleaved must reside, or at least pass through, the same compartment as the proteinase, (2) the cleavage is not always independent of the context, (3) the cleavage may be incomplete and unpredictable, (4) efficient cleavage will only be produced in cells actively expressing the proteinase, and (5) the post-translational cleavage is not compatible with all possible sub-cellular targetings. In many ways, a co-translational strategy such as reinitiation, which would be independent of cellular or viral factors, would be desirable. In reinitiation, ribosomes first translate an upstream cistron, although highly inefficiently, then resume translation of the second, downstream, cistron. Reinitiation was indeed tried in the 1980s, but the large imbalance makes it unsuitable for co-expression of even two genes (reviewed in [1]).

### The foot-and-mouth disease virus (FMDV) 2A sequence as a co-expression tool

Picornaviruses, the same family of viruses to first provide the IRESs, encode all their proteins in a long single ORF that is cleaved post-translationally by viral proteinases. However, it was described in the 1980's that at one position, the polyprotein of some picornaviruses (such as FMDV) underwent a rapid co-translational self-processing. It was soon realised that the key was a small 18aa peptide (2A) that directed its own separation from the growing polyprotein. During the last decade, this mechanism has been studied in detail, resulting in a simple model: the small 2A peptide, during its translation, interacts with the exit tunnel of the ribosome to induce the "skipping" of the last peptide bond at the C-terminus of 2A. The crucial point is that the ribosome is able to continue translating the downstream gene, after releasing the first protein fused in its C-terminus to 2A (reviewed in [5]). This type of sequence has been termed CHYSEL (cis-acting hydrolase element). From a biotechnological standpoint, all that is needed is to clone the coding sequence of 2A, followed by the codon for the first amino acid of the next FMDV protein (2B), in frame between the two genes one wishes to co-express. The synthesis of the peptide bond between the last amino acid (Gly) of 2A and the first (Pro) of 2B is skipped, producing an upstream protein with a C-terminal tail of 18aa (2A) and a downstream protein with a Pro at the N-terminus. The extra sequences have minimal effect on the activity of most proteins and none on their stability. In fact, the 2A peptide has been used as an efficient tag for immunoprecipitation and Western blotting, although commercial antibodies are not yet available. Interestingly, additional CHYSEL sequences have been found in viruses other than FMDV (for a review of these "2A-like" sequences, see [5]).

### Broad applicability of 2A

The initial publications using this strategy have shown that 2A skipping can be used in the typical viral vectors used for gene therapy (retrovirus and adeno-associated virus) to reliably co-express many reporter proteins (neomycin phosphotransferase, NEO; puromycin N-acetyl transferase, PAC; green fluoresecent protein, GFP, etc) and therapeutic proteins (*Herpes simplex *virus-1 thymidine kinase, HSV1TK; interleukin-12, IL-12; viral antigens, etc.) in transient transduced or stable cells lines and in animals. A full list of publications using 2A is available on the web [6]. Several publications in the past few months have shown the potential of this new co-expression strategy [7-9].

### Co-ordinating the co-expression of all your genes

**Figure 1:**
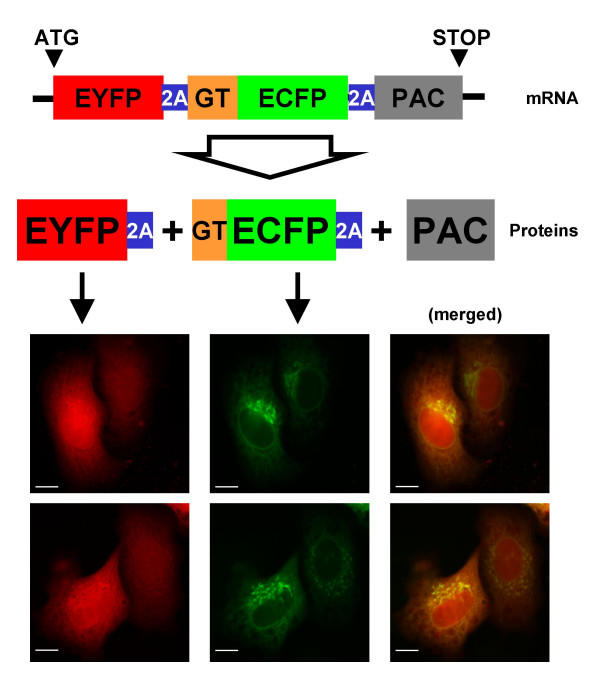
**Co-ordinated co-expression to different compartments in HeLa cells. **A single ORF was designed with the fluorescent genes *eyfp *and *ecfp *plus the puromycin resistant gene *pac *[9]. These genes were cloned flanking FMDV 2A sequences. An internal signal-anchor from the human β-1,4 galactosyltransferase (GT) was fused to the 5' end of the *ecfp *for Golgi targeting. During its translation, the self-processing of this polyprotein produced EYFP-2A that diffused to the cytoplasm and nucleus (due to its small size), while GT-EYFP-2A was co-translationally targeted to the Golgi apparatus by the GT signal (some protein also stays in the endoplasmic reticulum, due to the continuous cycling between these compartments). Two fields are shown, in both cases the cell on the left shows a high level of expression of both proteins that were expressed at lower levels in the cell on the right, illustrating the co-ordination obtained with the 2A co-expression strategy. PAC was able to confer resistance to puromycin. Images were taken 48 hours post-transfection. Bar represents 10 μm.

Up to four genes have been successfully co-expressed from plasmids and retrovirus using several copies of the FMDV 2A or other 2A-like sequences (to avoid direct repeats in retroviruses) [8,9]. Not only was co-expression effective, its co-ordination was also apparent [7,9] (Fig. 1), and the imbalance in the level of the proteins expressed was low (determined to 1.2 [8]). These properties allowed polycistronic vectors bearing *pac *in the last position to easily generate stable cell lines co-expressing two upstream genes [7,9].

### Putting your proteins where they should be

The CHYSEL strategy of co-expression is also compatible with the most disparate sub-cellular localisations [7-10]. Proteins processed by 2A from polyproteins were targeted to the cytosol, nucleus, mitochondria, endoplasmic reticulum, Golgi apparatus, plasma membrane (both, by transmembrane proteins and by cytosolic attachment due to myristoylation) and the extra-cellular compartment. Post-translationally targeted cytosolic proteins as well as co-translationally secreted and transmembrane proteins type I, II and III, have been successfully co-translated. Only one combination of co-translational signals was not correctly targeted [9].

### Designing complex polyproteins for multi-gene deficiency

**Figure 2:**
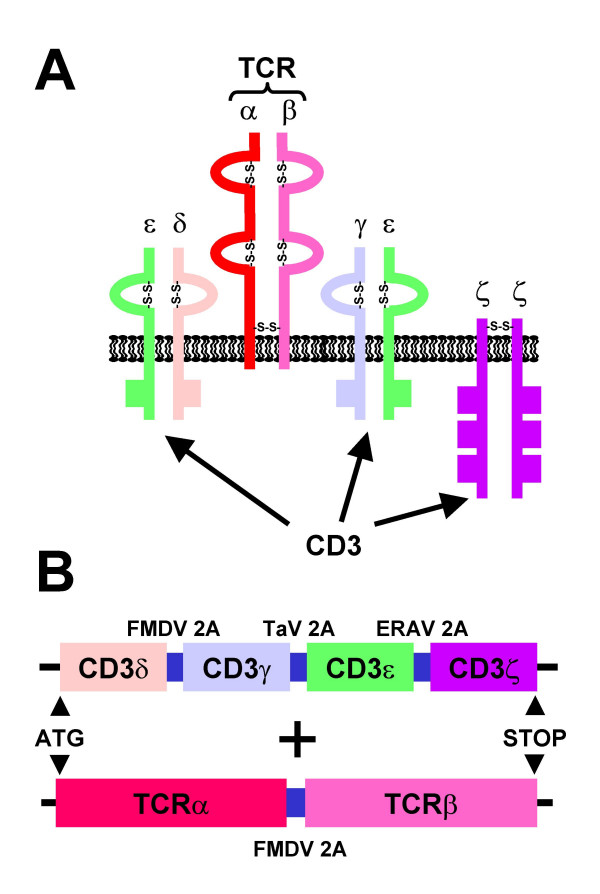
**Self-processing polyproteins to reconstruct the TCR:CD3 complex. (A) **Schematic diagram of the TCR:CD3 complex spanning the cytoplasmic membrane. The T-cell receptor (TCR) is formed by two subunits and the other four proteins assemble in three dimers to form the CD3 complex. The square boxes in the cytoplasmic sequences of the CD3 subunits represent the immunoreceptor tyrosine-based activation motifs (ITAMs). **(B) **To express the TCR:CD3 complex in cells, two retroviral vectors were designed to carry the two ORFs drawn here [8]. In the retrovirus encoding the four CD3 subunits, three different 2A sequences were used to avoid deletions due to direct repetitions.

The results reported in reference [8] should be particularly interesting for researchers in the gene therapy field. They provide a good example of the potential of the 2A co-expression strategy, introducing up to four genes in a single vector. Furthermore, they show the utility of this strategy to reconstruct a very delicate heteromultimeric protein complex on the cell surface (T-cell receptor:CD3 complex, TCR:CD3; Fig. 2A). It is known that all six subunits are necessary for the efficient formation of the TCR:CD3 complex and just two retroviral vectors were sufficient to reconstruct it in transfected 293T or infected 3T3 cells: one encoding both subunits of the T-cell receptor and the other the four subunits of the CD3 complex (Fig. 2B).

Lethally irradiated CD3ε^ΔP/ΔP ^× CD3ζ ^-/- ^mice (lacking all four CD3 subunits) were transplanted with bone marrow from wt C57BL/6 mice or CD3ε^ΔP/ΔP ^× CD3ζ ^-/- ^mice transduced with a retrovirus encoding the four CD3 subunits, and in both cases TCR surface expression was detected and the T cells proliferated normally after immune stimulation. Bone marrow from CD3ε^ΔP/ΔP ^× CD3ζ ^-/- ^mice without CD3 transduction did not restore T-cell development. T cells were also reconstituted in sub-lethally irradiated RAG-1^-/- ^mice (lacking mature T and B lymphocytes) in which bone marrow from CD3ε^ΔP/ΔP ^mice (lacking CDε and with a severe inhibition of CD3γ and CD3δ), transduced with a retrovirus encoding these three subunits (via two 2A sequences), was used for a transplant into the RAG-1^-/- ^mice. The same experiment using three vectors encoding the CD3 subunits separately was unsuccessful.

## Conclusions

The development of FMDV 2A as a cloning tool is an example of how dangerous pathogenic viruses can be harnessed by biotechnology for human benefit. Their molecular "tricks" (as IRES or CHYSEL sequences) are gradually becoming part of the biotechnologists' toolbox. The development of the polycistronic vectors here discussed is a big step forward, a decade and a half after the launching of the very first gene therapy trial with the aim of introducing in blood cells just a single therapeutic gene, adenosine deaminase (ADA), and the NEO marker [11]. These results represent a considerable advance in the correction of diseases that involve heteromultimeric proteins, several enzymes involved in a biochemical pathway or various proteins for combined/synergistic effects. 2A is not a magic tool that is going to solve all our problems, but it will help to pave the way for gene therapy.

## Competing interests

None declared.
